# Comparative speed of kill of sarolaner (Simparica^™^) and afoxolaner (NexGard^®^) against induced infestations of *Amblyomma americanum* on dogs

**DOI:** 10.1186/s13071-016-1378-8

**Published:** 2016-02-19

**Authors:** Robert H. Six, William R. Everett, Sara Chapin, Sean P. Mahabir

**Affiliations:** Zoetis, Veterinary Medicine Research and Development, 333 Portage St, Kalamazoo, MI 49007 USA; BerTek, Inc., PO Box 606, Greenbrier, AR 72058 USA

**Keywords:** *Amblyomma americanum*, Tick, Dog, Simparica™, Sarolaner, NexGard®, Afoxolaner, Isoxazoline, Oral, Speed of kill

## Abstract

**Background:**

The lone star tick*, Amblyomma americanum*, infests dogs and cats in North America and is the vector of the pathogens that cause monocytic and granulocytic ehrlichiosis in dogs and humans. A parasiticide’s speed of kill is important to minimize the direct and deleterious effects of tick infestation and especially to reduce the risk of transmission of tick-borne pathogens. In this study, speed of kill of a novel orally administered isoxazoline parasiticide, sarolaner (Simparica^™^ chewable tablets), against *A. americanum* on dogs was evaluated and compared with afoxolaner (NexGard^®^) for 5 weeks following a single oral dose.

**Methods:**

Based on pretreatment tick counts, 24 dogs were randomly allocated to treatment with sarolaner (2 to 4 mg/kg), afoxolaner (2.5 to 6.8 mg/kg) or a placebo. Dogs were examined and live ticks counted at 8, 12, and 24 h after treatment and subsequent re-infestations on Days 7, 14, 21, 28, and 35. Efficacy was determined at each time point relative to counts for placebo dogs.

**Results:**

A single oral dose of sarolaner provided 100 % efficacy within 24 h of treatment, and consistently provided >90 % efficacy against subsequent weekly re-infestations with ticks to Day 28. Significantly more live ticks were recovered from afoxolaner-treated dogs than from sarolaner-treated dogs at 24 h after infestation from Day 7 through Day 35 (*P* ≤ 0.0247). At 24 h, efficacy of afoxolaner declined to less than 90 % from Day 14 to the end of the study. There were no adverse reactions to treatment.

**Conclusions:**

In this controlled laboratory evaluation, sarolaner had a faster speed of kill against *A. americanum* ticks than afoxolaner. The rapid and consistent kill of ticks by sarolaner within 24 h after a single oral dose over 28 days, suggests this treatment will provide highly effective and reliable control of ticks over the entire treatment interval, and could help reduce the risk of transmission of tick-borne pathogens by *A. americanum*.

## Background

Ticks are viewed by pet owners and veterinarians as both a nuisance and a threat; heavy and prolonged tick infestations can cause anemia, especially in young or small dogs [1], and they are vectors of pathogens of domestic and wild animals, as well as of people [2]. The lone star tick, *Amblyomma americanum,* is the vector of *Ehrlichia chaffeensis* and *Ehrlichia ewingii* [3, 4], to dogs and man, which cause monocytic and granulocytic ehrlichiosis, respectively. *Borrelia lonestari*, a probable cause of erythema migrans in man, has also been detected in *A. americanum* ticks using DNA amplification techniques [5]. *Amblyomma americanum* is one of the most common tick species infesting dogs and cats in North America. Its geographic range has expanded from the southern states, across the southern plains, through the Midwest and into the eastern states. Focal populations have also been reported in northern states including Maine, New York, Massachusetts, Connecticut, and New Jersey [6, 7].

Tick prevention and control have taken on a new importance as the awareness of, and exposure to, tick-borne diseases increases and *A. americanum* (and other tick species) populations continue to expand. Topically administered parasiticides with contact activity have been the most common approach to tick control on the dog, but recently a new class of compounds, the isoxazolines, have demonstrated efficacy against ticks for 1 month or longer following a single oral dose [8, 9]. One of these, afoxolaner, has been reported to provide ≥98.9 % efficacy against *A. americanum* for up to 30 days after a single dose when assessed at 72 h after infestation [10]. Sarolaner is a novel isoxazoline which in a chewable tablet formulation (Simparica^™^) provides excellent control of fleas and ticks for at least 1 month after a single oral dose (TL McTier, personal communications), and has demonstrated >90 % efficacy against *A. americanum* within 48 h after re-infestation for at least 28 days [11].

The speed of acaricidal activity is critical in disrupting or preventing feeding of ticks and thus reducing the risk of pathogen transmission which generally occurs after the infected tick is attached and feeding for at least 24 to 48 h [12, 13], though recently transmission of *Ehrlichia canis* by *Rhipicephalus sanguineus* has been shown to occur within as little as 3 h after attachment [14]. A laboratory study was conducted to evaluate and compare the speed of kill of sarolaner (Simparica^™^) and afoxolaner (NexGard^®^) against existing *A. americanum* infestations and weekly re-infestations for a period of 5 weeks following treatment with a single dose.

## Methods

### Ethical approval

The study was a masked, negative controlled, randomized laboratory efficacy design conducted in Arkansas, USA. Study procedures were in accordance with the World Association for the Advancement of Veterinary Parasitology (WAAVP) guidelines for evaluating the efficacy of parasiticides for the treatment, prevention and control of flea and tick infestation on dogs and cats [15], and complied with the principles of Good Clinical Practices [16]. The protocol was reviewed and approved by the local Institutional Animal Care and Use Committee. Masking of the study was assured through the separation of functions. All personnel conducting observations or animal care or performing infestations and counts were masked to treatment allocation.

### Animals

Fourteen male and 10 female, purpose-bred Beagles (14) and mixed breed dogs (10) from 8 to 30 months of age and weighing from 6.7 to 13.1 kg were used in the study. Each dog was individually identified by a unique ear tattoo or electronic transponder and had undergone an adequate wash-out period to ensure that no residual ectoparasiticide efficacy remained from any previously administered treatments. Dogs were individually housed in indoor runs such that no physical contact was possible between them and were acclimatized to these conditions for at least 14 days prior to treatment. Dogs were fed an appropriate maintenance ration of a commercial dry canine feed for the duration of the study. Water was available *ad libitum*. All dogs were given a physical examination to ensure that they were in good health at enrollment and suitable for inclusion in the study. General health observations were performed twice daily throughout the study.

### Design

The study followed a randomized complete block design. Dogs were ranked according to decreasing tick counts into blocks of three and within each block a dog was randomly allocated to treatment with either a placebo, sarolaner or afoxolaner. There were eight dogs per treatment group. Dogs were infested with ticks 2 days prior to treatment and then weekly for 5 weeks. Tick counts were conducted at 8, 12, and 24 h after treatment and each subsequent weekly re-infestation. During the study it was noted that two afoxolaner-treated dogs had been incorrectly dosed: one underdosed, receiving approximately half the label dose, and the other overdosed with about double the label dose. As the misdosing was expected to bias the tick counts for these two dogs, they were excluded from study analyses, thus there were only six evaluable dogs in the afoxolaner group. The uneven group sizes were incorporated into the statistical model.

### Treatment

Bodyweights taken on Day -2 were used to determine the appropriate dose to be administered. On Day 0, dogs received a placebo tablet, the appropriate strength sarolaner chewable tablet (Simparica^™^) to provide sarolaner at the recommended dose of 2 mg/kg (range 2 to 4 mg/kg), or NexGard^®^ per label directions (afoxolaner at 2.5 to 6.8 mg/kg). All doses were administered by hand pilling to ensure accurate and complete dosing. Each dog was observed for several minutes after dosing for evidence that the dose was swallowed, and for general health at 1, 4, and 24 h after treatment administration.

### Tick infestation and assessment

The ticks were obtained from the Oklahoma State University’s *A. americanum* colony which was initiated in 1976 with engorged females collected locally in Stillwater, OK. The colony has been maintained with the introduction of locally collected, engorged females every 2 years. The most recent introduction was approximately 1 year before the study was initiated.

Tick infestations were performed on Days -7 (host suitability and allocation), -2, 7, 14, 21, 28, and 35. At each infestation, a pre-counted aliquot of 50 (±5) viable unfed adult *A. americanum* were directly applied to the dog, which was then confined in an appropriately sized travel crate for approximately 4 h to restrict movement and facilitate tick attachment. Each dog was examined to remove and count live ticks at 48 h after the initial host suitability infestation. At 8 and 12 (±1) hours after treatment and each subsequent weekly re-infestation, the dogs were examined systematically so that the entire body surface was carefully examined and live ticks were counted *in situ*. At 24 (±1) hours after treatment and each subsequent weekly re-infestation, the dogs were examined and then thoroughly combed to count and remove live ticks. Each dog was examined for at least 10 min. If ticks were encountered in the last minute, combing was continued in 1 min increments until no ticks were encountered.

### Statistical analysis

The individual dog was the experimental unit and the primary end point was live tick counts. Data for post-treatment live (free plus attached) tick counts were summarized with arithmetic (AM) and geometric (GM) means by treatment group and time point. Tick counts were transformed by the log_e_ (count + 1) transformation prior to analysis in order to stabilize the variance and normalize the data. Using the PROC MIXED procedure (SAS 9.2, Cary NC), transformed counts were analyzed using a mixed linear model. The fixed effects were treatment, time point and the interaction between time point and treatment by time point. The random effects included room, block within room, block by treatment interaction and error. Testing was two-sided at the significance level α = 0.05.

The assessment of efficacy for live ticks was based on the percent reduction in the arithmetic and geometric mean live tick counts relative to placebo and to the positive control, as suggested by the most recent guidelines of the WAAVP for systemic acaricides [13] and was calculated using Abbott’s formula:

$$ \%\ \mathrm{reduction}=100 \times \frac{\mathrm{mean}\ \mathrm{count}\ \left(\mathrm{placebo}\right)\hbox{--} \mathrm{mean}\ \mathrm{count}\ \left(\mathrm{treated}\right)}{\mathrm{mean}\ \mathrm{count}\ \left(\mathrm{placebo}\right)} $$

## Results

There were no treatment-related adverse events during the study. Placebo-treated dogs maintained good tick infestations throughout the study with mean tick counts ranging from approximately 11 to 28 (Tables 1, 2 and 3).

**Table 1:** Mean live *Amblyomma americanum* counts and efficacy relative to placebo at 8 h after treatment and post-treatment re-infestations for dogs treated with a single oral dose of sarolaner or afoxolaner on Day 0^1^

Treatment		Day of treatment or re-infestation					
		0	7	14	21	28	35
Placebo	Range	4–21	12–32	6–26	11–34	13–36	5–22
	A. mean	11.6	21.9	19.1	20.1	26.3	16.5
	G. mean^2^	10.5^a^	20.8^a^	17.7^a^	18.9^a^	24.9^a^	15.0^a^
Sarolaner	Range	5–16	14–24	8–20	11–22	8–34	9–27
	A. mean	9.3	19.5	13.0	16.5	17.5	17.3
	Efficacy (%)	20.4	10.9	32.0	18.0	33.3	0.0
	G. mean^2^	8.7^a^	19.1^a^	12.5^a^	16.0^a^	15.9^a^	16.3^a^
	Efficacy (%)	16.8	8.4	29.5	15.5	36.0	0.0
	*P*-value vs. placebo	0.4573	0.7074	0.1450	0.4773	0.0595	0.7298
Afoxolaner	Range	5–22	8–28	5–21	6–28	8–41	12–26
	A. mean	14.7	19.3	12.8	17.3	22.0	18.2
	Efficacy (%)	0.0	11.6	32.9	13.9	16.2	0.0
	G. mean^2^	13.0^a^	17.9^a^	11.4^a^	15.1^a^	19.0^a^	17.2^a^
	Efficacy (%)	0.0	14.3	35.5	20.1	23.7	0.0
	*P*-value vs. placebo	0.4465	0.5668	0.1130	0.4092	0.3137	0.6098
	*P*-value vs. sarolaner	0.2185	0.8293	0.7795	0.8560	0.5722	0.8543

^1^ *n* = 6 for afoxolaner, *n* = 8 for placebo and sarolaner groups

^2^Geometric means within columns with the same superscript are not significantly different (*P* > 0.05)

**Table 2:** Mean live *Amblyomma americanum* counts and efficacy relative to placebo at 12 h after treatment and post-treatment re-infestations for dogs treated with a single oral dose of sarolaner or afoxolaner on Day 0^1^

Treatment		Day of treatment or re-infestation					
		0	7	14	21	28	35
Placebo	Range	4–20	13–33	7–27	11–34	15–36	5–26
	A. mean	12.4	21.5	20.1	20.9	26.9	18.4
	G. mean^2^	11.2^a^	20.5^a^	18.9^a^	19.5^a^	25.7^a^	16.6^a^
Sarolaner	Range	0–16	9–22	9–18	10–23	8–34	12–28
	A. mean	3.8	15.0	12.6	16.9	16.9	18.4
	Efficacy (%)	69.7	30.2	37.3	19.2	37.2	0.0
	G. mean^2^	1.9^b^	14.4^a^	12.4^a^	16.2^a^	15.5^b^	17.6^a^
	Efficacy (%)	82.8	29.6	34.4	17.0	39.6	0.0
	*P*-value vs. placebo	<0.0001	0.1392	0.0791	0.4291	0.0338	0.8025
Afoxolaner	Range	0–15	8–27	8–21	9–25	8–41	12–21
	A. mean	6.2	17.7	13.7	16.7	21.8	17.5
	Efficacy (%)	50.2	17.8	32.1	20.2	18.8	4.8
	G. mean^2^	4.1^b^	16.4^a^	12.6^a^	15.4^a^	18.9^a,b^	16.8^a^
	Efficacy (%)	63.9	20.1	33.2	21.3	26.6	0.0
	*P*-value vs. placebo	0.0008	0.4068	0.1427	0.3790	0.2500	0.9633
	*P*-value vs. sarolaner	0.0657	0.6850	0.9533	0.8665	0.5317	0.8806

^1^ *n* = 6 for afoxolaner, *n* = 8 for placebo and sarolaner groups

^2^Geometric means within columns with the same superscript are not significantly different (*P* > 0.05)

**Table 3:** Mean live *Amblyomma americanum* counts and efficacy relative to placebo at 24 h after treatment and post-treatment re-infestations for dogs treated with a single oral dose of sarolaner or afoxolaner on Day 0^1^

Treatment		Day of treatment or re-infestation					
		0	7	14	21	28	35
Placebo	Range	4–22	14–31	7–33	9–36	17–36	9–26
	A. mean	14.1	21.1	22.3	22.0	28.4	20.3
	G. mean^2^	12.6^a^	20.2^a^	20.1^a^	19.9^a^	27.4^a^	19.1^a^
Sarolaner	Range	0–0	0–0	0–2	0–2	0–9	1–20
	A. mean	0.0	0.0	0.5	0.4	3.6	8.0
	Efficacy (%)	100	100	97.8	98.3	87.2	60.5
	G. mean^2^	0.0^b^	0.0^c^	0.4^c^	0.3^c^	2.5^c^	5.5^b^
	Efficacy (%)	100	100	98.2	98.7	91.1	70.9
	*P*-value vs.placebo	<0.0001	<0.0001	<0.0001	<0.0001	<0.0001	<0.0001
Afoxolaner	Range	0–0	0–4	0–11	3–16	9–29	10–23
	A. mean	0.0	1.5	4.5	9.8	17.7	18.0
	Efficacy (%)	100	92.9	79.8	55.3	37.7	11.1
	G. mean^2^	0.0^b^	0.9^b^	3.0^b^	8.5^b^	16.1^b^	17.0^a^
	Efficacy (%)	100	95.3	84.9	57.6	41.5	10.6
	*P*-value vs. placebo	<0.0001	<0.0001	<0.0001	0.0025	0.0487	0.6801
	*P*-value vs. sarolaner	0.9525	0.0247	0.0003	<0.0001	<0.0001	0.0007

^1^ *n* = 6 for afoxolaner, *n* = 8 for placebo and sarolaner groups

^2^Geometric means within columns with the same superscript are not significantly different (*P* > 0.05)

At the 8-h time point, tick counts for both products were not significantly different from placebo-treated dogs or each other (*P* ≥ 0.0595) at any evaluation with percent reductions ranging from 0 to 36 % (GM) (Table 1).

At the 12-h time point, sarolaner-treated dogs had significantly lower tick counts than placebo-treated dogs (*P* ≤ 0.0338) on Days 0 and 28, with efficacies (GM) of 82.8 % and 39.6 %, respectively (Table 2). Treatment with afoxolaner resulted in significantly lower tick counts than placebo at 12 h on Day 0 only (*P* = 0.0008) with efficacy (GM) of 63.9 %. The tick counts were similar for sarolaner and afoxolaner-treated dogs on all count days (*P* ≥ 0.0657).

**Fig. 1:**
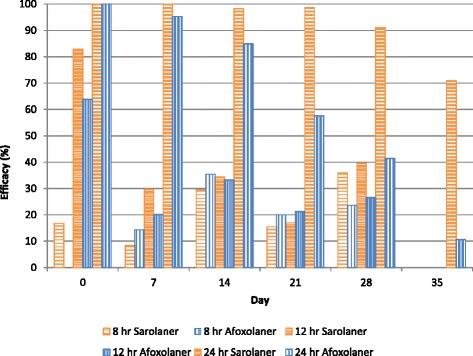
Percent efficacy based on geometric mean counts relative to placebo at 8, 12, and 24 h after treatment and weekly post-treatment re-infestations of *Amblyomma americanum* for dogs treated with a single oral dose of sarolaner or afoxolaner on Day 0

At the 24-h time point, dogs treated with sarolaner had significantly lower tick counts than placebo-treated dogs (*P* < 0.0001) from treatment to Day 35, and these counts were also lower than those for afoxolaner-treated dogs (*P* ≤ 0.0247) at all post-treatment re-infestations (Days 7 to 35) (Table 3). Afoxolaner-treated dogs had significantly lower tick counts than placebo from Day 0 to Day 28 (*P* ≤ 0.0487). Treatment with sarolaner resulted in efficacy of at least 91.1 % (GM) to Day 28, while efficacy for dogs treated with afoxolaner declined below 90 % from Day 14 onwards (Table 3, Fig. 1).

## Discussion

A single dose of sarolaner resulted in the rapid reduction of an existing infestation of *A. americanum* ticks and the rapid kill of tick re-infestations after treatment. Efficacy (GM) of >90 % was achieved within 24 h for 28 days. Rapid kill of ticks is important to reduce the direct adverse effects of tick feeding and is critical in the reduction of tick-borne pathogen transmission. Thus, the rapid efficacy of a single oral dose of sarolaner shown in this study should provide a marked reduction in the risk of a treated dog becoming infected with the pathogens transmitted by *A. americanum*. However, further studies directly examining the effect of treatment on the transmission and infectivity of these individual pathogens are needed to confirm the levels of protection provided through sarolaner’s acaricidal efficacy.

Efficacy of afoxolaner against *A. americanum* for its one month label claim was determined at 72 h after re-infestations and efficacy of ≥97.8 % (GM) was attained for up to 30 days after treatment [10]. Efficacy of tick control products is generally recommended to be assessed at 48 h after re-infestation [15]. A relatively slower speed of kill of *A. americanum* for afoxolaner was apparent in the current study, where efficacy at 24 h declined to below 85 % (GM) from Day 14 onwards. By contrast, treatment with sarolaner resulted in significantly superior reductions in tick counts to afoxolaner against post-treatment re-infestations to Day 35 (*P* ≤ 0.0247) and efficacy at 24 h was >90 % (GM) through Day 28.

## Conclusions

This study confirmed the acaricidal efficacy of sarolaner against *A. americanum* after a single oral administration at the label dose. Ticks were killed rapidly, with the majority of ticks (>90 %) killed within 24 h after weekly re-infestations for at least 4 weeks. Sarolaner’s speed of kill was consistently higher than that of afoxolaner from Day 7 onwards at 24 h. Sarolaner chewable tablets (Simparica^™^) offers the pet owner and veterinarian an efficacious oral product with a rapid speed of kill of lone star ticks over the entire month following a single oral dose, making it an important new tool for the treatment and prevention of tick infestation with the potential to reduce the risk of tick-borne pathogen transmission.

## References

[1] Baker CF, Hunter JS, McCall JW, Young DR, Hair JA, Everett W (2011). Efficacy of a novel topical combination of fipronil, amitraz and (S)-methoprene for treatment and control of induced infestations with four North American tick species (*Dermacentor variabilis*, *Ixodes scapularis*, *Amblyomma americanum* and *Amblyomma maculatum*) on dogs. Vet Parasitol..

[2] Sonenshine DE, Sonenshine DE (1991). The female reproductive system. Biology of Ticks. Vol. I.

[3] Beall MJ, Alleman AR, Breitschwerdt EB, Cohn LA, Couto CG, Dryden MW (2012). Seroprevalence of *Ehrlichia canis*, *Ehrlichia chaffeensis* and *Ehrlichia ewingii* in dogs in North America. Parasit Vectors.

[4] Blagburn BL, Dryden MW (2009). Biology, treatment and control of flea and tick infestations. Vet Clin Small Anim..

[5] James AM, Liveris D, Wormser GP, Schwartz I, Montecalvo MA, Johnson BJ (2001). *Borrelia lonestari* infection after a bite by an *Amblyomma americanum* tick. J Infect Dis..

[6] Childs JE, Paddock CD (2003). The ascendancy of *Amblyomma americanum* as a vector of pathogens affecting humans in the United States. Annu Rev Entomol..

[7] Merten HA, Durden LA (2000). A state-by-state survey of ticks recorded from humans in the United States. J Vector Ecol..

[8] Rohdich N, Roepke RKA, Zschiesche E (2014). A randomized, blinded, controlled and multi-centered field study comparing the efficacy and safety of Bravecto^®^ (fluralaner) against Frontline^®^ (fipronil) in flea- and tick-infested dogs. Parasit Vectors..

[9] Shoop WL, Harline EJ, Gould BR, Waddell ME, McDowell RG, Kinney JB (2014). Discovery and mode of action of afoxolaner, a new isoxazoline parasiticide for dogs. Vet Parasitol..

[10] FDA. FOI. Supplemental NADA 141-406. NEXGARD. http://www.fda.gov/downloads/animalveterinary/products/approvedanimaldrugproducts/foiadrugsummaries/ucm409102.pdf. Accessed 20 Nov 2015.

[11] Six RH, Everett WR, Young DR, Carter L, Mahabir SP, Honsberger NA, et al. Efficacy of a novel oral formulation of sarolaner (Simparica^™^) against five common tick species infesting dogs in the United States. Vet Parasitol. http://dx.doi.org/10.1016/j.vetpar.2015.12.023 10.1016/j.vetpar.2015.12.023 26935819

[12] Little SE. Changing paradigms in understanding transmission of canine tick-borne diseases: the role of interrupted feeding and intrastadial transmission. In: 2nd Canine Vector-Borne Disease (CVBD) Symposium. Mezara del Vallo, Sicily, Italy. 2007; pp. 30-4. http://www.cvbd.org/fileadmin/media/cvbd/Proceedings_CVBD_2007_FINAL23042007.pdf

[13] Salinas LJ, Greenfield RA, Little SE, Voskuhl GW (2010). Tick borne infections in the southern United States. Am J Med Sci..

[14] Fourie JJ, Stanneck D, Luus HG, Beugnet F, Wijnveld M, Jongejan F (2013). Transmission of *Ehrlichia canis* by *Rhipicephalus sanguineus* ticks feeding on dogs and on artificial membranes. Vet Parasitol..

[15] Marchiondo AA, Holdsworth PA, Fourie LJ, Rugg D, Hellmann K, Snyder DE (2013). World Association for the Advancement of Veterinary Parasitology (W.A.A.V.P.) second edition: Guidelines for evaluating the efficacy of parasiticides for the treatment, prevention and control of flea and tick infestations on dogs and cats. Vet Parasitol.

[16] EMEA. Guideline on good clinical practices. VICH Topic GL9. http://www.ema.europa.eu/docs/en_GB/document_library/Scientific_guideline/2009/10/WC500004343.pdf. Accessed 23 Aug 2015.

